# ASO Author Reflections: Expanding the Role of Targeted Axillary Dissection in Patients with Higher Nodal Burden After Neoadjuvant Systemic Therapy

**DOI:** 10.1245/s10434-026-20176-4

**Published:** 2026-07-09

**Authors:** A. J. T. Lee, A. Kwong

**Affiliations:** Queen Mary Hospital, The University of Hong Kong, Pok Fu Lam, Hong Kong

## Past

For many years, the key issue in postneoadjuvant axillary surgery has been how to balance oncologic accuracy with surgical morbidity. The development of targeted axillary dissection improved the accuracy of nodal restaging by combining removal of the originally biopsied positive node with sentinel lymph node biopsy, but most evidence has come from patients with limited nodal disease.^1–3^ The current clinically important question is whether this strategy remains dependable in patients with higher nodal burden, because a substantial proportion of node-positive patients achieve axillary pathological complete response after neoadjuvant systemic therapy. Our study was designed to address this specific gap by examining the performance of targeted axillary dissection in patients with advanced nodal disease.

## Present

Our findings suggest that targeted axillary dissection can be highly accurate even in patients with more advanced nodal involvement. In this prospective cohort, technical success was high, clipped-node retrieval was consistently achieved, and no false-negative cases were observed amongst the evaluable patients. These results are clinically meaningful, because they indicate that a higher initial nodal burden does not necessarily preclude safe surgical de-escalation provided the original involved node is accurately marked and removed. Equally important, nearly half of the patients achieved axillary pathologic complete response, highlighting the potential to spare many women the morbidities of formal axillary dissection. In a field where overtreatment remains a concern, our data support a broader and more thoughtful application of targeted axillary dissection in carefully selected node-positive patients.

## Future

The next step is to validate these findings in larger, multicenter populations with longer follow-up. Future research should help define which patients with higher nodal burden are best suited for this approach. Standardization of technical aspects, including marking methods, tracer use, and definition of procedural success, will be important to improve reproducibility across institutions. Longer-term outcome data are also needed to determine whether omission of axillary dissection after a negative targeted axillary dissection is associated with durable regional control and acceptable survival in this patient cohort. If these findings are confirmed, axillary surgery may move further towards a more individualized approach, in which treatment intensity is guided by tumor response rather than initial nodal burden alone.

## References

[1] Caudle AS, Yang WT, Krishnamurthy S, et al. Improved axillary evaluation following neoadjuvant therapy for patients with node-positive breast cancer using selective evaluation of clipped nodes: implementation of targeted axillary dissection. *J Clin Oncol*. 2016;34(10):1072–8. 10.1200/JCO.2015.64.0094 PMC4933133 26811528

[2] Donker M, Straver ME, Wesseling J, et al. Marking axillary lymph nodes with radioactive iodine seeds for axillary staging after neoadjuvant systemic treatment in breast cancer patients: the MARI procedure. *Ann Surg*. 2015;261(2):378–82. 10.1097/SLA.0000000000000558 24743607

[3] Simons JM, van Nijnatten TJA, van der Pol CC, et al. Diagnostic accuracy of radioactive iodine seed placement in the axilla with sentinel lymph node biopsy after neoadjuvant chemotherapy in node-positive breast cancer. *JAMA Surg*. 2022;157(11):991–9. 10.1001/jamasurg.2022.3907 PMC9453629 36069889

